# Morphological analysis of human umbilical vein endothelial cells co-cultured with ovarian cancer cells in 3D: An oncogenic angiogenesis assay

**DOI:** 10.1371/journal.pone.0180296

**Published:** 2017-07-03

**Authors:** Xiao Wan, Phurit Bovornchutichai, Zhanfeng Cui, Eric O’Neill, Hua Ye

**Affiliations:** 1 CRUK/MRC Oxford Institute for Radiation Oncology, Department of Oncology, Medical Sciences Division, University of Oxford, Oxford, Oxfordshire, United Kingdom; 2 Institute of Biomedical Engineering, Department of Engineering Science, Mathematical, Physical and Life Sciences Division, University of Oxford, Oxford, Oxfordshire, United Kingdom; University of Sheffield, UNITED KINGDOM

## Abstract

Antiangiogenic therapy for cancer is a strategy targeted at tumour vasculature, often in combination with conventional cytotoxicity treatments. Animal testing is still the most common method used for evaluating the efficacy of new drugs but tissue-engineered *in vitro* models are becoming more acceptable for replacing and reducing the use of animals in anti-cancer drug screening. In this study, a 3D co-culture model of human endothelial cells and ovarian cancer cells was developed. This model has the potential to mimic the interactions between endothelial cells and ovarian cancer cells. The feasibility of applying this model in drug testing was explored here. The complex morphology of the co-culture system, which features development of both endothelial tubule-like structures and tumour structures, was analysed quantitatively by an image analysis method. The co-culture morphology integrity was maintained for 10 days and the potential of the model for anti-cancer drug testing was evaluated using Paclitaxel and Cisplatin, two common anti-tumour drugs with different mechanisms of action. Both traditional cell viability assays and quantitative morphological analyses were applied in the drug testing. Cisplatin proved a good example showing the advantages of morphological analysis of the co-culture model when compared with mono-culture of endothelial cells, which did not reveal an inhibitory effect of Cisplatin on the tubule-like endothelial structures. Thus, the tubule areas of the co-culture reflected the anti-angiogenesis potential of Cisplatin. In summary, *in vitro* cancer models can be developed using a tissue engineering approach to more closely mimic the characteristics of tumours *in vivo*. Combined with the image analysis technique, this developed 3D co-culture angiogenesis model will provide more reproducible and reliably quantified results and reveal further information of the drug’s effects on both tumour cell growth and tumour angiogenesis.

## Introduction

Oncogenic angiogenesis, the formation of new capillary networks formed by vascular endothelial cells, is crucial in tumour development for the provision of nutrients and oxygen to a growing tumour [1–2]. The endothelium in these newly-formed blood vessels can also promote tumour development via paracrine pathways and influence the effects of chemotherapy [3–4]. Pre-clinical models have been developed to evaluate potential candidates for regulating oncogenic angiogenesis. Among these models, Engelbreth-Holm-Swarm (EHS) extracellular matrix (ECM) extract or Matrigel, is one of the most frequently used matrices in *in vitro* angiogenesis models. The high levels of laminin and growth factors within Matrigel promote vascular endothelial cells to form tubule-like structures [5]. However, this is a short-term assay, because the tubule structures formed by HUVECs degrade after 48 hours [6] whereas in the *in vivo* environment the angiogenesis process would take place in a few days. Another limitation is that the well-established Matrigel-based angiogenesis assay is based on culture of endothelial cells on their own, thus lacking the intercellular interactions between the endothelium and cancer cells *in vivo*. Three-dimensional co-culture of endothelial cells with cancer cells in extracellular matrix was designed by other researchers to address the above problems [3–4, 6–8]. Nevertheless, despite keeping EHS extracellular matrix as the main component in the culture matrix, most of the time the tubule formation ability of endothelial cells was lost [4]. Conventionally, tubule-like endothelial structures were formed when HUVECs were cultured on top of a thin layer of Matrigel [6, 8]. In addition, despite the importance of image analysis for 3D *in vitro* models, many previous reports did the processing of the images manually [7]. Some attempts have been made to process the images semi-automatically and automatically [9–11], but application to the co-culture of endothelial cells with cancer cells in 3D culture is still required. The aim of this study, therefore, was to develop a 3D *in vitro* co-culture model for cancer angiogenesis studies to test drugs with anti-angiogenesis potential. This co-culture model features both a vascular network and tumour structures in a 3D environment with an imaging technique (i.e. image segmentation utilised to provide quantitative results), without the need for further fluorescence labelling.

## Materials and methods

### Cell and drug solution preparation

With the exception of Cisplatin and Palictaxel, which were from Sigma-Aldrich, US, all the chemicals were from Thermo-Fisher, UK. Human ovarian cancer cell line OVCAR8 was a kind gift from Dr. Richard Callaghan (Nuffield Department of Clinical Laboratory Sciences, University of Oxford). The cancer cells were cultured in high-glucose Dulbecco's Modified Eagle Medium (DMEM, Lonza, UK) supplemented with 10% (v/v) foetal bovine serum (FBS, Life Technologies, US) and 1% (v/v) penicillin (100U/mL)-streptomycin (100μg/mL) (PAA, US). Human umbilical vein endothelial cells (HUVECs, Lonza, UK) were cultured in endothelial growth medium (EGM-2, Lonza, UK) and passages 2–6 were used for the study. HUVECs older than P6 were discarded as they lost tube formation ability.

### 3D sandwich co-culture of OVCAR8 with HUVECs in Matrigel

**Fig 1:**
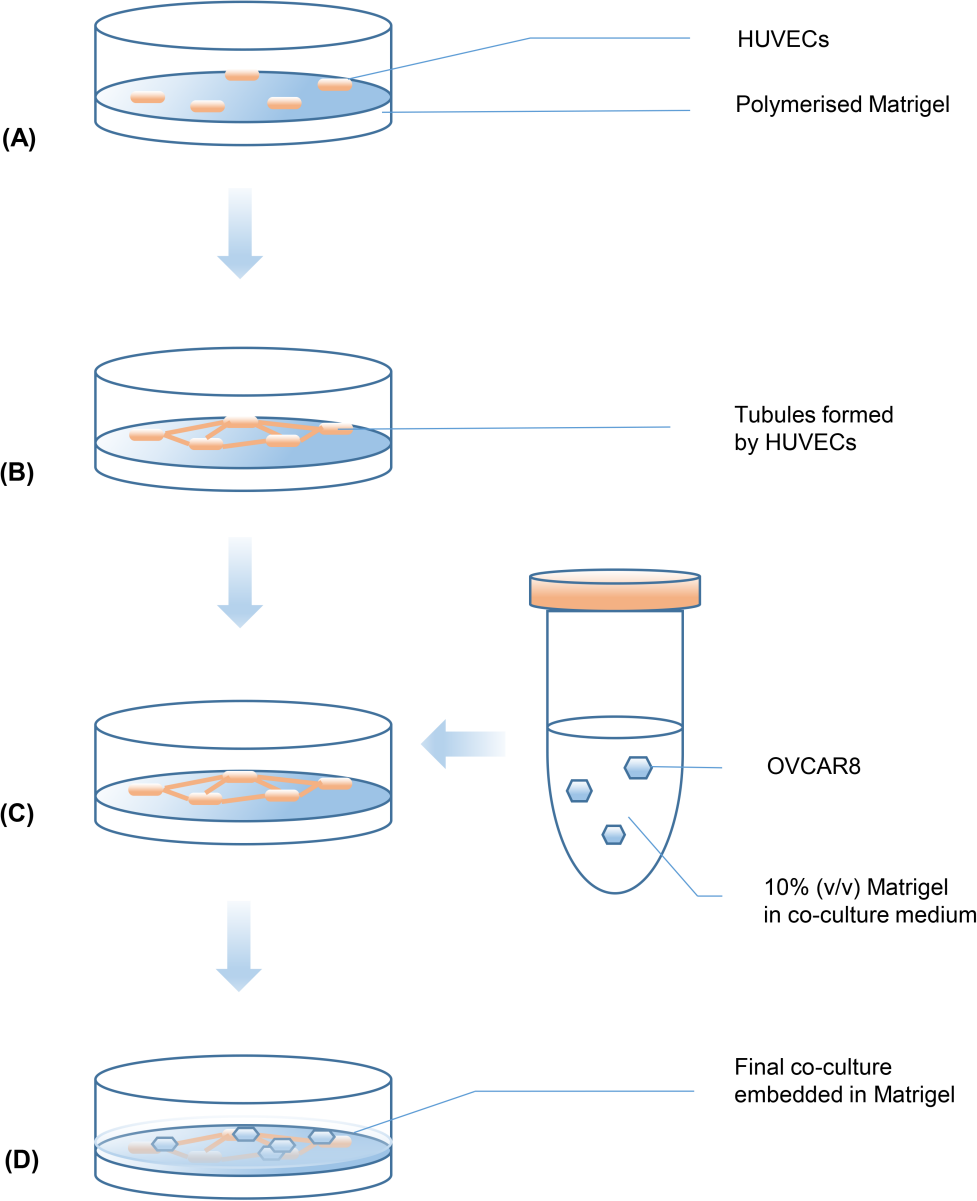
Schematic procedure for 3D co-culture of HUVECs and OVCAR8 in Matrigel sandwich. (A) HUVECs seeded on polymerised Matrigel; (B) After 4 hours, HUVECs started to form tubules; (C) OVCAR8 cell suspension in medium containing 10% Matrigel was added; (D) 24 hours later, Matrigel sandwich structure formed and co-culture stabilised, ready for longer term culture or further drug testing.

A 3D culture protocol based on the Matrigel sandwich structure was adapted from previous research [12] and is illustrated in Fig 1. Briefly, to form the bottom layer of Matrigel, 120μL pure Matrigel was added into each well of a 24-well plate (NUNC) which had been pre-chilled on ice. The plates were then incubated at 37^°^C for 30min, allowing the Matrigel to polymerise. Then 3.5×10^4^ HUVECs cells suspended in 250μL endothelial basic medium (EBM-2, Lonza, UK) supplemented with 2% FBS (Life Technologies, US) and 1% penicillin-streptomycin (PAA) (Fig 1(A)) were seeded onto the polymerised gel layer. After 4 hours of HUVECs seeding, 1.25×10^4^ OVCAR8 cells in 250μL pre-chilled EBM-2 containing 10% Matrigel (v/v), supplemented with 2% FBS and 1% penicillin-streptomycin were added onto the polymerised Matrigel (Fig 1(B)). The plate was then left at 37^°^C to allow the top layer of Matrigel to polymerise (Fig 1(C)). The co-culture system was maintained in EBM-2 supplemented with 2% FBS and 1% penicillin-streptomycin at 37^°^C, 5% CO_2_ for at least 24 hours before further drug testing (Fig 1(D)).

### 3D sandwich mono-cultures of OVCAR8 or HUVECs in Matrigel

The process was the same as the procedure described in co-culture. The only difference was that no HUVECs were added, so OVCAR8 cells were cultured alone in an overall 175μL of Matrigel. For HUVECs mono-culture, the process was the same as the procedure described in co-culture except that no OVCAR8 cells were added following the seeding of HUVECs, so HUVECs were cultured alone in an overall 175μL of Matrigel.

### 3D co-culture of OVCAR8 with HUVECs on top of Matrigel

The process was the same as the procedure described in sandwich 3D co-culture, except that no top layer of Matrigel was added. As a result, HUVECs and cancer cells were cultured on top of the 120 μL bottom layer of Matrigel without a sandwich-embedded structure.

### Image segmentation and analysis using MATLAB

Images were initially pre-processed in order to correct a non-uniform illumination using a polynomial background correction technique [13]. This consisted of a series of entropy thresholding, image erosion and image dilation which allowed the background to be separated from the cells. The outcome of this image analysis workflow is explained in detail in the Results section.

### Immunofluorescence staining for Matrigel-based 3D cultures

For immunofluorescence, cells in 3D culture were stained using a protocol modified for sandwich Matrigel [12]. The cultures were first rinsed by PBS-glycine (100mM glycine in PBS) three times, then fixed with 4% formaldehyde (Thermo Fisher, UK). Blocking buffer (10% goat serum (Sigma), 1% goat F(ab’)2 anti-mouse immunoglobulin G (Caltag, UK) in staining buffer (PBS supplemented with 0.2% TritonX-100, 0.1% BSA, and 0.05% Tween 20) was used to block non-specific binding sites. The samples were then incubated with the primary antibody (mouse IgG2B anti-human vWF A2 domain (R&D, US); rabbit anti-human cleaved caspase– 3 (CST, UK)) at 4^°^C overnight, rinsed twice with PBS, and then incubated with the second antibody (rabbit anti-mouse IgG labelled with TRITC (Sigma-Aldrich, US); goat anti-rabbit IgG labelled with Alexa 633) at 4^°^C for 1 hour. Then the samples were washed once with staining solution and once with PBS and then diaminophenylindole (DAPI) hard-set mounting medium (Vector Labs, UK) was applied. 3D constructs for images were obtained from a modified BioRad Radiance 2100 MP Multiphoton Microscope (MPM, Zeiss; Jena, Germany). The emission filters selected were: 495 nm for blue, 525 nm for green and 595 nm for red. Serial optical sections were then loaded into Imaris 7.6.1 (Bitplane Ag, Zurich, Switzerland) software for processing and analysis.

### AlamarBlue assay for cell viability testing

For IC50 estimation for a specific model to a certain drug, AlamarBlue (Life Technologies, US) cell viability testing was conducted based on the instructions from the manufacturer. Cells were incubated with EBM-2 containing 10% AlamarBlue for 2 hours at 37^°^C. The fluorescence intensity was measured at 560 nm excitation and 590 nm emission using a fluorescent micro-plate reader (WALLAC VICTOR2 1420 multilabel counter model, Perkin Elmer, UK). The inhibition percentage of anti-cancer drug on the viability of cells was calculated as below: Percentage of Inhibition = [cell viability of untreated sample–cell viability of sample treated by anti-cancer drug]/ cell viability of untreated sample ×100%. The log-dose dependent responses were then fitted into Sigmoidal functions by GraphPad Prism (GraphPad Software, US) to calculate IC50 estimation.

### Statistics

All experiments were repeated three times with triplicates, if not stated otherwise. Student’s *t*-test was used to compare parameters between different groups. *P<0*.*05* was considered to be significantly different.

## Results

### Co-culture of HUVECs and cancer cells allowed maintenance of tubule structures for 10 days

**Fig 2:**
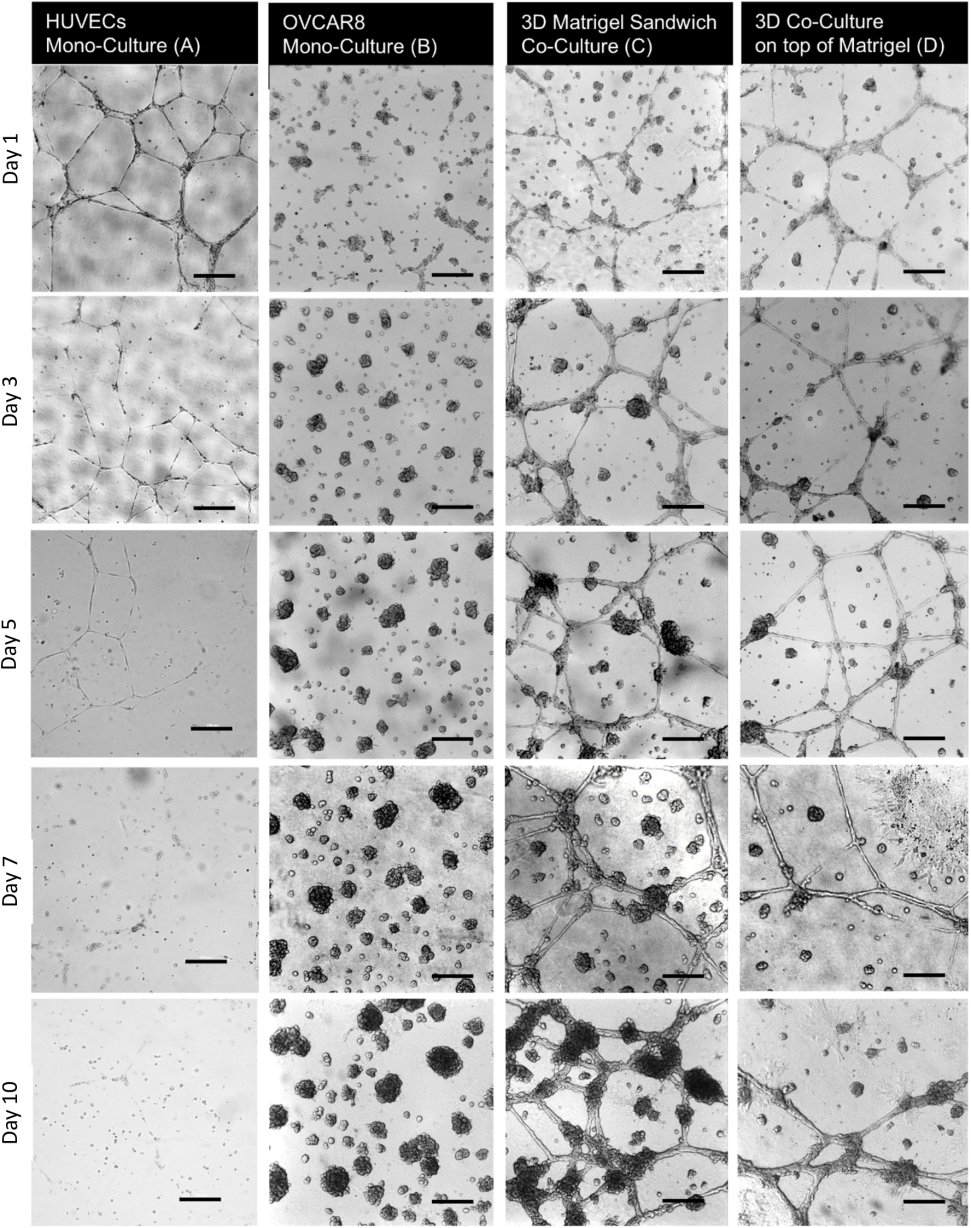
Morphology characterisation of different *in vitro* models used in the study. (A) Representative pictures of (A) HUVECs in mono-culture, (B) OVCAR8 in mono-culture, (C) 3D co-culture of both cell types in the Matrigel sandwich, and (D) on-top co-culture of both cell types on top of the Matrigel at day 1 (24 hours after seeding on day 0), day 3, day 5, day 7 and day 10. It is noticeable that the tubule structures of HUVECs mono-culture started to degrade at day 3.

**Fig 3:**
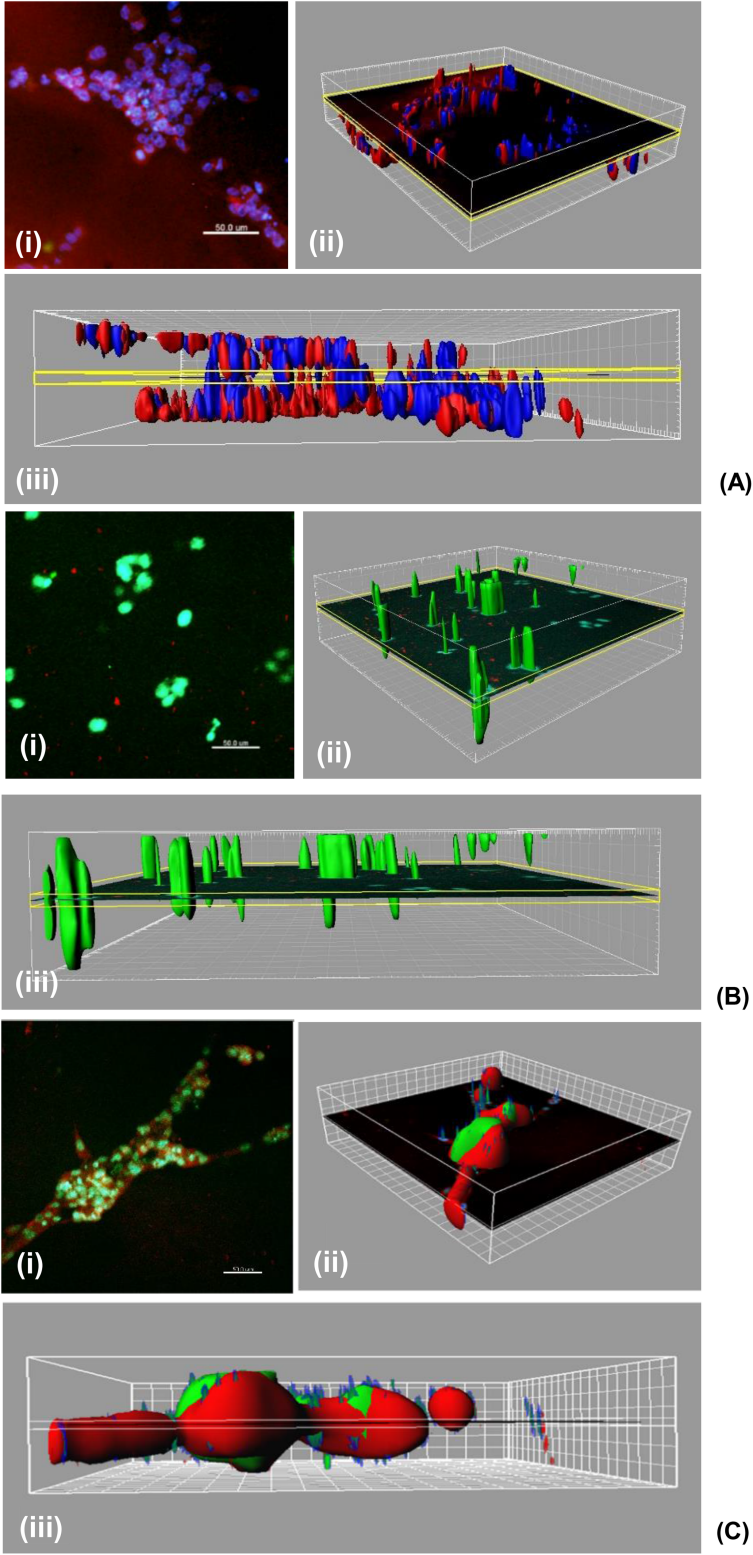
Immunostaining characterisation of 3D Matrigel sandwich models used in the study. 3D structure analysis by multiphoton microscopy (MPM). Green: GFP-expressing OVCAR8; Red: vWF staining of HUVECs by TRITC; Blue: Cell nuclei marked by DAPI. Scale bar: 200μm. (A) HUVECs 3D mono-culture in the Matrigel sandwich, at day 2 (from day 3 most tubule structure degraded); (B) OVCAR8 3D mono-culture in the Matrigel sandwich at day 10; (C) Co-culture of HUVECs and OVCAR8 in the Matrigel sandwich at day 10. (A)–(C): (i) original fluorescence photo; (ii) 3D reconstruction by Imaris; (iii) Side view of the 3D reconstruction showing the thickness. In the 3D reconstruction figure the grid unit measurement is 20μm.

In sandwich co-culture, a combination of tubules and spheroids was observed, as shown in Fig 2(C), and these structures were maintained and kept growing for 10 days. This relatively long culture time is in contrast to the fact that the tubule network formed by HUVECs cultured alone in the Matrigel sandwich showed degradation at day 2 and there were no signs of living cells at day 10 (Fig 2(A)). On the other hand, OVCAR8 cultured alone in the Matrigel sandwich formed spheroid structures and kept growing for 10 days. It is shown that OVCAR8 cells cultured in EBM-2 did not show different morphology or growth rate when compared to those cultured in their normal growth medium DMEM (S1 Fig). Compared with co-culture, the morphology of OVCAR8 cultured alone in the Matrigel sandwich had no obvious tubule network formed (Fig 2(B)). The different morphology between 3D co-culture and 3D mono-culture indicated that the tubule network structure shown in the 3D co-culture was formed under the influence of the co-culture and could not be formed in mono-culture of OVCAR8. Interestingly, as the tubule structures formed by HUVECs in mono-culture already degraded at day 10 (Fig 2(A)), we had to question whether or not the tubule network was mainly formed by OVCAR8 only at day 10. The multiphoton microscopy imaging shown in Fig 3 clarified that there were still HUVECs existing in the tubule structures at day 10, marked using the endothelial cell-specific molecule vWF labelled by red fluorescent TRITC, while cancer cell OVCAR8 expressed green fluorescence protein (GFP). From the MPM image in Fig 3, it was confirmed that after long-term co-culture, HUVECs were still not overtaken by cancer cells. OVCAR8 were adhering to the tubule structure formed by HUVECs in the form of 3D spheroid structures. The grid frame in the reconstructed 3D image in Fig 3 showed that the co-culture system had an average thickness of around 200 μm, implying that the two types of cells had formed a 3D structure in the Matrigel, based on the fact that diameters of a single cell of HUVECs and OVCAR8 are both around 20–60 μm.

### Semi-automated morphology analysis comparing different models

**Fig 4:**
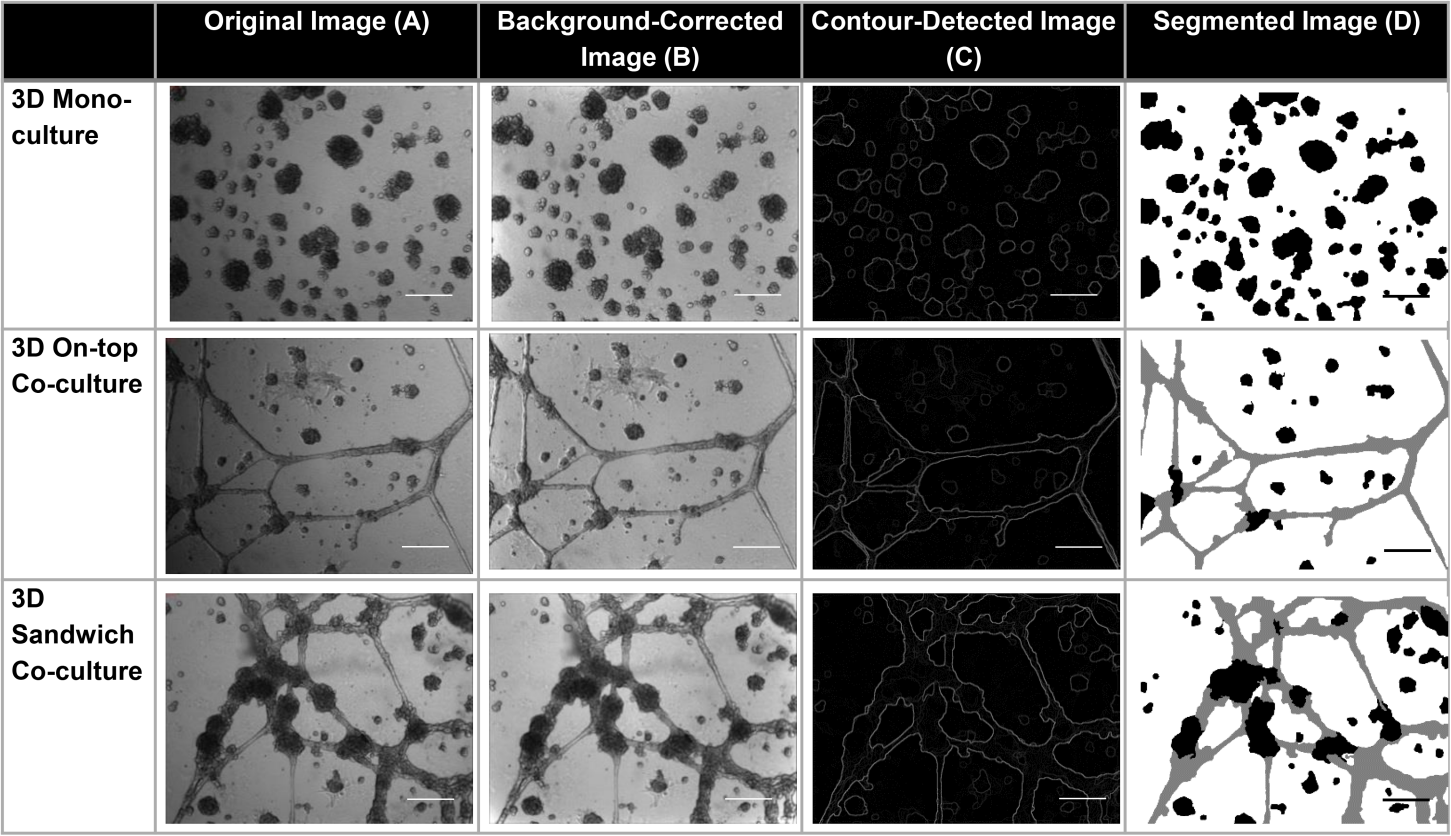
Image processing procedure used for bright field images. (A) The original bright field images of 3D mono-culture of OVCAR8 cells, on-top co-culture of OVCAR8 cells and HUVECs, and 3D co-culture of OVCAR8 cells and HUVECs at day 10. (B) Background corrected images of each original image. (C) Different contour lines detected in each background corrected image. (D) Segmentation of spheroids (in black colour) and tubules (in grey colour) in each image.

The images were originally taken from the microscope at 3,488 x 2,616 pixels. Their resolution was later reduced by 25% to 872 x 654 pixels in order to reduce the computation resource required for segmenting each image. The image was initially pre-processed to correct an uneven illumination in the background using the polynomial correction technique. Some of the images have an uneven background illumination which is caused by the cells being off-centre with respect to the light source of the microscope. Illumination of these images tends to run from dark to bright as shown in Fig 4A. Hence, the background correction using polynomial fitting is suitable for these images. To begin with, the background was separated from the cells (OVCAR8 spheroids and HUVECs) using the entropy filtering method in which the entropy of each pixel was calculated from its 9-by-9 neighbourhood [13]. The entropy values were then manually thresholded to create a binary mask containing the background and the cells. The resulting binary mask contained holes inside the cells which were filled using a morphological close operation with a disc-shaped structuring element with a radius of 8 pixels. The cells were then subtracted from the image using the binary mask, and the region of each cell in the image was filled with the pixel values on its boundary. This resulted in the background being extracted from the image. The extracted background was then least-square fitted to a 4th degree polynomial which gave an estimated background illumination as an output. This output was subtracted from the input image giving the image with corrected background illumination. This pre-processing step allowed the segmentation of cells to be more accurate as it created a sharper contrast between the cells and the background (Fig 4A and 4B).

Segmentation of the cells was performed using contour detection and hierarchical image segmentation technique [14]. This technique detects different contour intensities within the image. The contour intensities were then transformed into hierarchy of regions (Fig (4)). As most automated systems for tubule-structure segmentation mainly focused on monocultures [10–11] they were not feasible to be used for our data as there was more than one irregular-shaped type of cells. However, there were some reports concerned with co-cultures [15–16] but the challenges faced in our study were the fact that OVCAR8 and HUVECs tend to cluster together, especially in 3D co-cultures. This highlighted the difficulty of segmenting the two cells from one another as the cell morphologies of each type are not consistent. On top of that, the images were taken in greyscale, thus adding to the segmentation issues as both OVCAR8 and HUVECs were similar in intensity. In order to produce results that were accurate enough, the entire image analyses here were semi-automated.

After detecting the cells’ contours, OVCAR8 spheroids, HUVECs and the background were segmented from the image based on human-drawn annotations. The annotations were automatically propagated to nearby unlabelled regions resulting in user-specified segmentations. These were refined until high-quality segmentations of cells were yielded (Fig 4(D)). The processed images were then analysed with regard to angiogenesis-related parameters and spheroid characteristic parameters. Pixels classified as OVCAR8 spheroids and HUVECs were summed up and converted into the unit of μm^2^ to represent total spheroid area, which is the projection of the actual 3D spheroid structures. As the number of spheroids in each image was not consistent, the total areas were divided by the total number of spheroids to obtain an average spheroid area represented in each image. Nevertheless, the aim of image analyses in this study was not to create a software to automatically segment the cells i.e. OVCAR8 and HUVECs from each other and from the background, but rather to use them as a pipeline to assist the quantification of the respective cellular areas.

**Fig 5:**
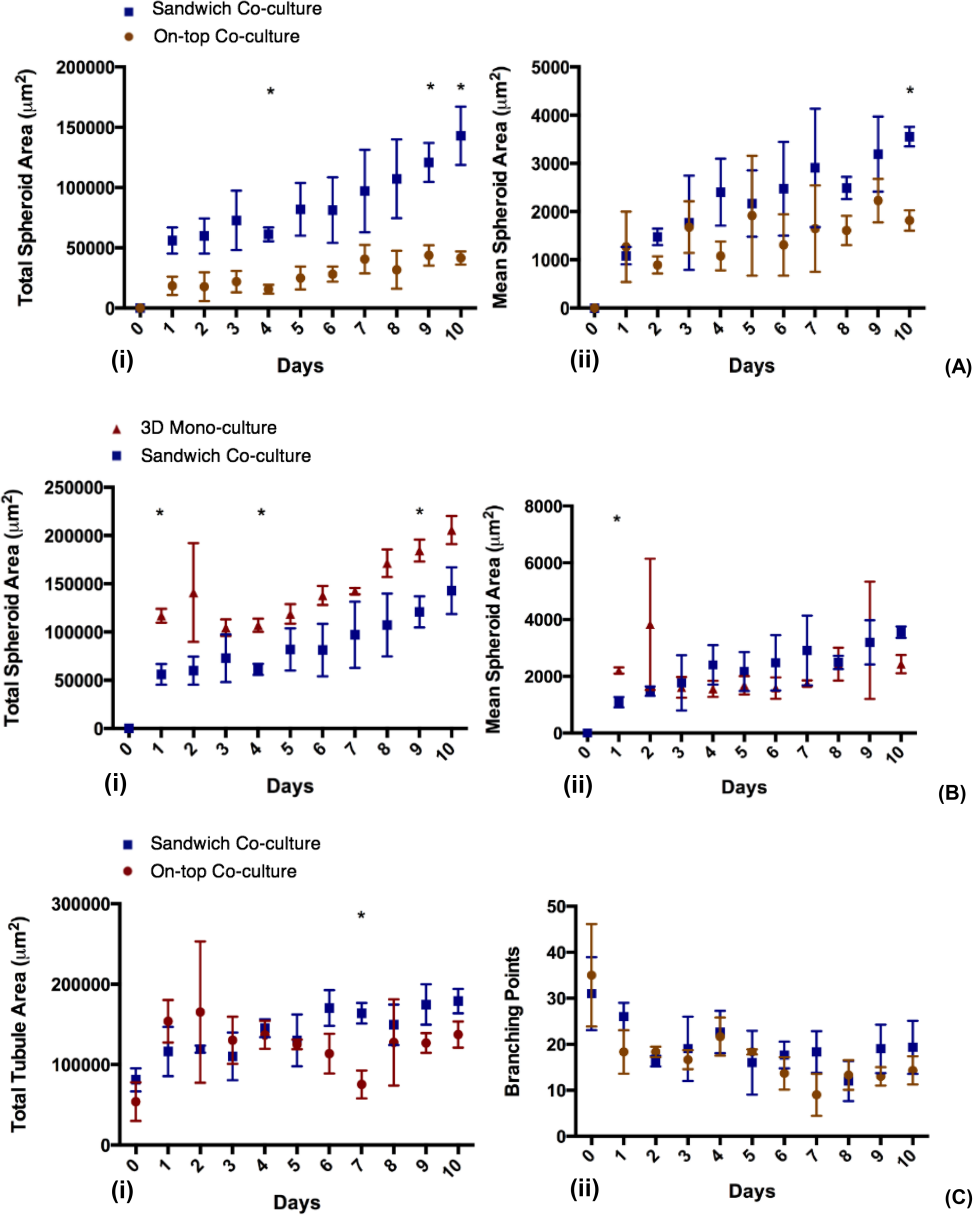
Semi-automated image analysis comparing morphologies of different *in vitro* models. (A) Comparison of 3D on-top and 3D sandwich co-culture of OVCAR8 and HUVECs with regard to: (i) Total spheroid areas in μm^2^ and (ii) Mean tubule areas. (B) Comparison of 3D mono-culture of OVCAR8 and 3D co-culture of HUVECs and OVCAR8 in terms of: (i) Total spheroid areas in μm^2^ and (ii) Mean spheroid areas in μm^2^. (C) Angiogenesis parameter comparisons for 3D on-top and 3D sandwich co-culture of OVCAR8 and HUVECs over 10 days for: (i) Total tubule areas and (ii) Branching points. Results are presented as (mean ± SD), and are considered significantly different when p < 0.05 based on Student’s t-test (marked as ‘*’ in the figures showing significant difference between the compared models on the same day). Note that since HUVECs mono-culture lost their tubule network at day 2 and eventually lost signs of living cells over longer term culture (Fig 2(A)), only mono-culture of cancer cell OVCAR8 and co-culture of OVCAR8 and HUVECs were compared here over 10 days of culture.

To see how a 3D sandwich structure influences the morphology of co-culture, comparisons of 3D on-top and sandwich co-cultures for 10 days were made in Fig 5(A). The results show a gradual increase in spheroid areas for both cultures over the culture period of 10 days. In Fig 5A (i), the total spheroid area for 3D co-cultures is greater than the spheroid area in 3D on-top co-cultures for the entire 10 days of culture. By calculating the mean spheroid area in each image, the spheroid area for 3D co-cultures (Fig 5A (ii)) was still greater than 3D on-top co-cultures for every single time point with the exception of the first day of culture. It is noticeable that the difference in spheroid areas between 3D and 3D on-top co-cultures became statistically significant towards the end of the culture period. After day 10, the total spheroid areas of the co-cultures were 142,902 μm^2^ for 3D and 41,617 μm^2^ for 3D on-top (p-value = 0.002), which were equivalent to the mean spheroid areas of 3558 μm^2^ for 3D and 1817 μm^2^ for 3D on-top co-cultures respectively (p-value = 0.0004).

To reveal the effects of HUVECs on the growth of cancer cell spheroids, spheroid areas from 3D co-cultures were compared to spheroid areas from 3D mono-cultures of OVCAR8 cells. Fig 5B (i) shows a gradual increase of total spheroid area over time for both cultures. Results showed that the total spheroid areas for 3D monocultures were greater than 3D co-cultures for the entire duration of the culture with statistically significant results shown during day 1,4 and 9 of the culture (with p-values = 0.001, 0.0009, and 0.005 respectively). Interestingly, comparisons between the mean spheroid areas in 3D monocultures and co-cultures showed a contradicting pattern of results. Fig 5B(ii) shows that the mean spheroid areas of monocultures were greater than the corresponding areas in co-cultures for only the first 3 days of culture with a statistically significant difference observed only in day 1. After day 3, the mean spheroid areas for co-cultures were shown to be greater than for mono-cultures. However, the difference between the cultures were not statistically significant.

For the angiogenesis analysis, representative different parameters (i.e. the total tubule areas and branching point numbers) were analysed and compared between the models. From the results shown in Fig 5C (i), fluctuations of total tubule area around 100,000 and 200,000 μm^2^ were observed throughout 10 days of culture for both 3D on-top and 3D sandwich co-cultures, with the exception of day 0. While the tubule areas of 3D on-top co-culture displayed a sinusoidal pattern, the total tubule area in 3D co-culture showed a consistent increase until it exceeded the corresponding result from day 6 onwards. At day 7, the total tubule area in 3D co-culture was significantly higher than 3D on-top co-culture (p-value = 0.002). The number of branching points for both 3D on-top and 3D sandwich co-culture images decreased over the first 2 days (Fig 5C (ii)). This can possibly be explained by the fusion of small tubules to create a larger tubule, facilitating better uptake of nutrients from the medium. This speculation is supported by the results from the plot of total tubule area (Fig 5C (i)) which showed an increase in tubule area over time. The number of branching points in 3D on-top and 3D sandwich co-culture images fluctuated from day 3 to day 6. The number of branching points in 3D co-cultures exceeded the ones in 3D on-top co-cultures from day 6 onwards. This observation was in conjunction with the results from the total tubule area, in which there was a divergence between 3D sandwich and 3D on-top co-culture results from day 6 onwards. However, the divergence of branching points between 3D on-top and sandwich cultures was not statistically significant.

**Fig 6:**
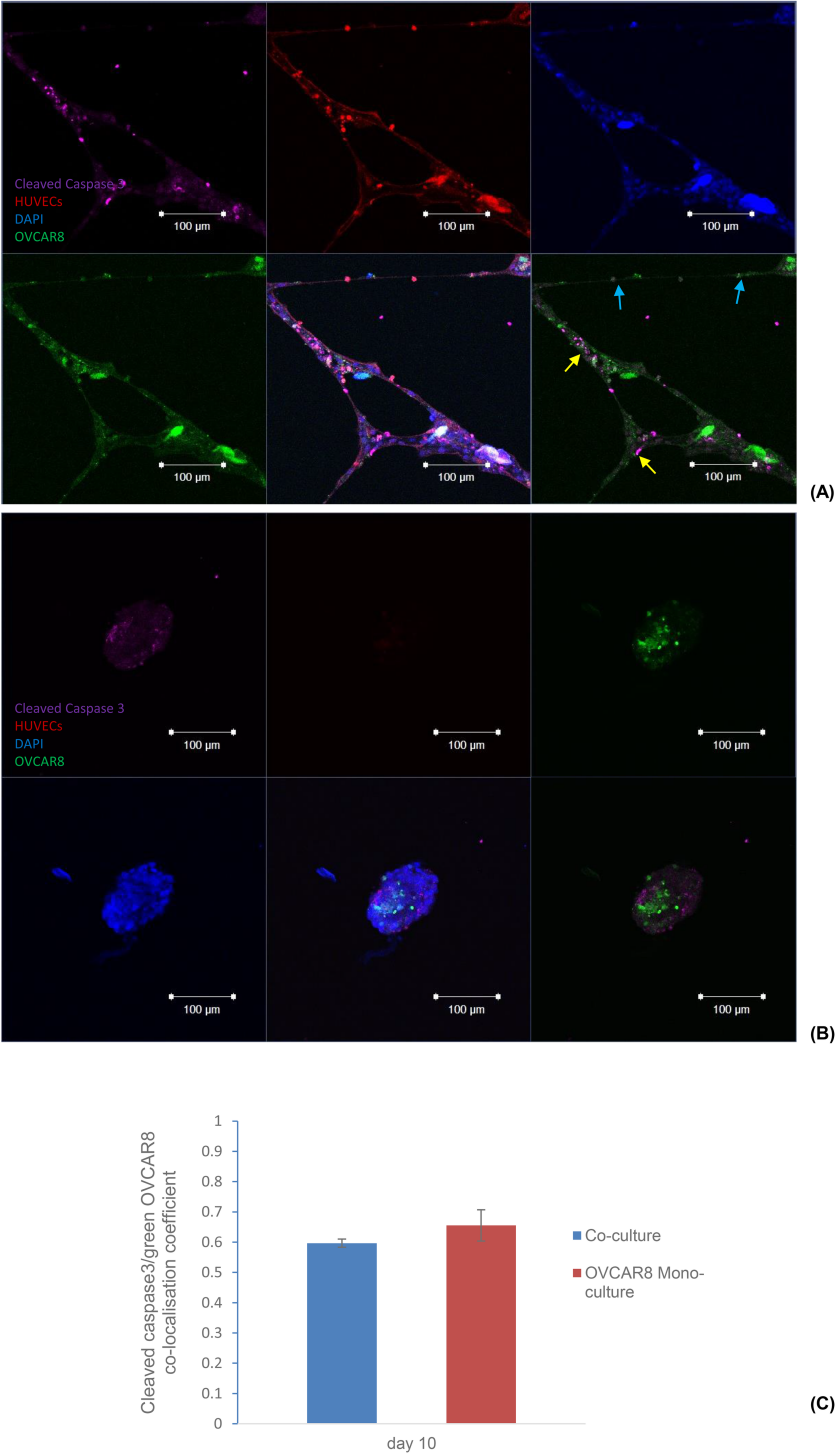
Apoptosis assay comparing co-culture of OVCAR8 with HUVECs and mono-culture of OVCAR8 at day 10. (A) Fluorescence images of co-culture. It is noticeable that nearly no apoptotic signal was observed with thinner tubule structures, while more apoptosis was detected with the structures with larger dimensions. (B) Fluorescent images of mono-culture. Cleaved caspase 3 (magenta); DAPI (blue), HUVECs (red), and OVCAR8 (green). Scale bar: 100μm. (C) Zen localisation coefficient comparison between co-culture and mono-culture. No significant difference was observed.

We also carried out an end-point assay (Fig 6) to see if apoptosis could be detected at day 10, and if co-culture with HUVECs could influence the apoptotic status of the cancer cells OVCAR8. We performed a co-localisation with ZEN image processing software (Zeiss, UK) of co-focal images from four parallel experiments and discovered, based the on ZEN co-localisation coefficient, that there seemed to be no significant difference between the percentages of apoptotic OVCAR8 cancer cells in the mono-culture when compared with the ones in the co-culture. However, a closer look at the original images revealed that in the co-culture, there was lower apoptotic signal in structures with thinner dimensions (indicated by the blue arrows in Fig 6(A)), while more apoptosis was observed in co-culture structures with larger dimensions (indicated by the yellow arrows in Fig 6(A)) which was possibly due to the inefficient nutrient and/or oxygen supplies to the central area of these structures. We therefore propose that there was a possibility that HUVECs were protecting cancer cells from apoptosis.

### Potential application of the developed sandwich co-culture model for drug screening

**Fig 7:**
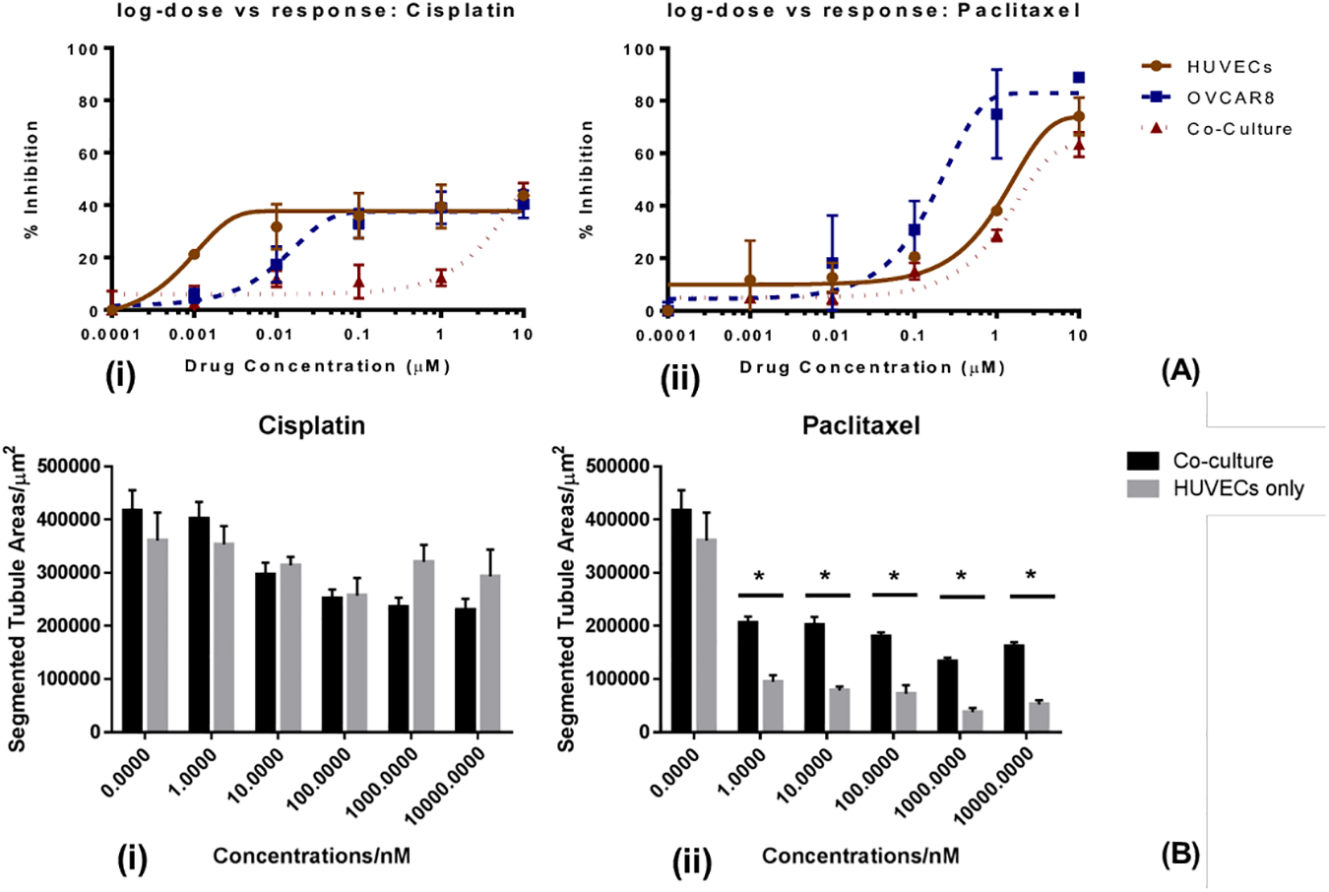
Drug response comparisons between 3D mono-cultures and co-cultures. (A) Dose-dependent responses comparing monocultures and co-culture, measured by cell viability inhibition percentages in a Matrigel sandwich treated with: (i) Cisplatin; (ii) Paclitaxel. (B) Tubule area reduction comparing monocultures and co-culture, after treatment by: (i) Cisplatin and (ii) Paclitaxel. *p<0.05 **p<0.01 compared with control using Student’s t-test.

**Fig 8:**
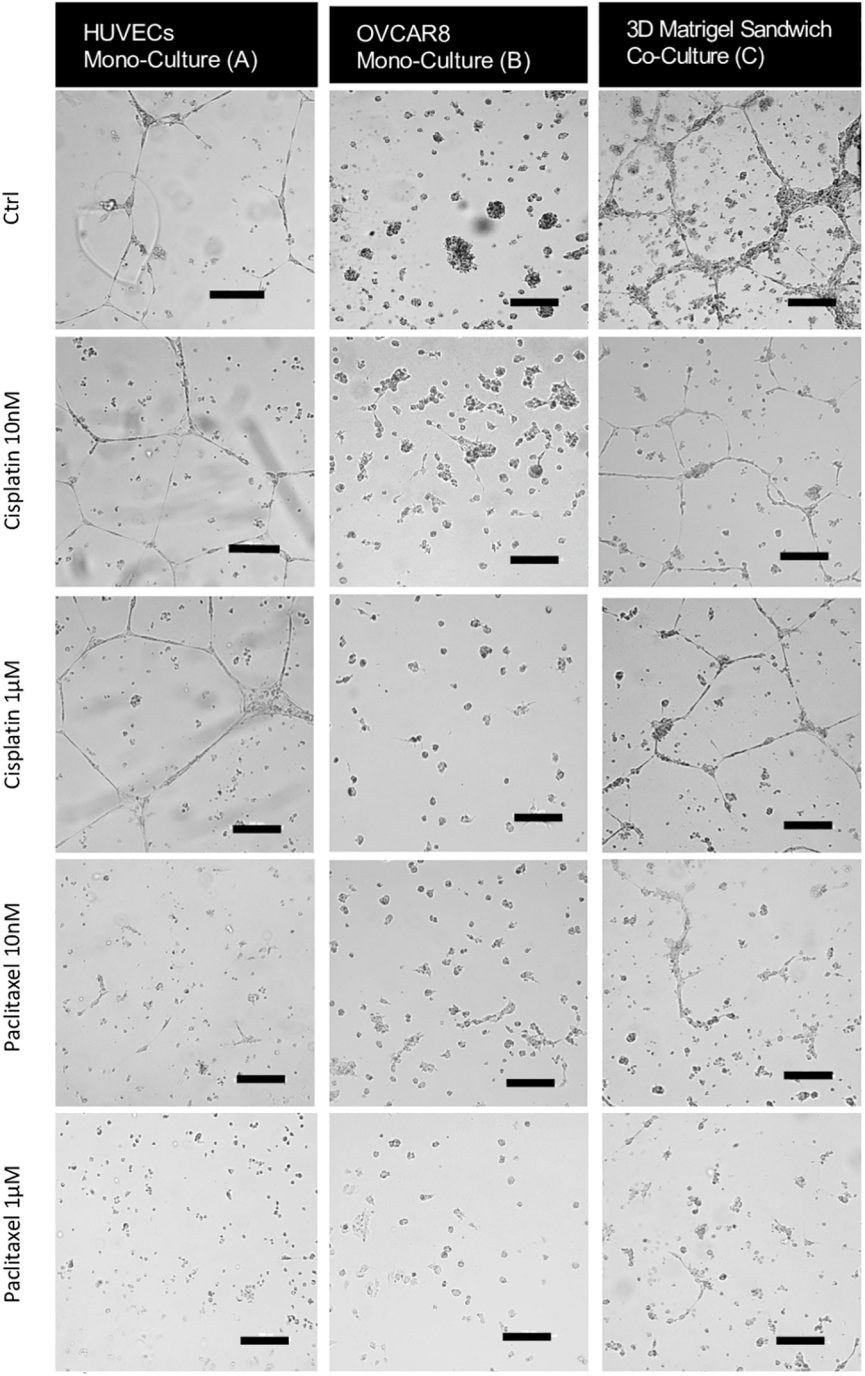
Representative images of the co-culture model and mono-culture models in response to Cisplatin or Paclitaxel. (A) Mono-culture of HUVECs; (B) Mono-culture of OVCAR8; (C) Co-culture of HUVECs and OVCAR8 in a Matrigel sandwich. The images at the concentrations of 0, 10nM, and 1μM are given as representatives, as most differences were observed between 10nM and 1μM. Scale bar: 200μm.

Sigmoid function was fitted to dose-dependent cell viability inhibition caused by the anti-cancer drugs Cisplatin and Paclitaxel. Both Cisplatin and Paclitaxel had lower inhibition effect on the co-culture model compared with mono-cultures of a single cell type. In the case of Cisplatin, the log-dose dependent response plotted in Fig 7(A) shows a clear higher drug resistance of co-culture compared with both mono-cultures over all the concentrations tested, but the Sigmoid curve fitting process was interrupted and failed to give an IC50 based on the provided data because the highest concentration (10μM) failed to give a >50% inhibition effect in all of the three models (HUVECs mono-culture, OVCAR8 mono-culture and co-culture of both cell types). For Paclitaxel, a higher drug resistance, or a lower drug response, was also observed in the co-culture when compared with the two mono-cultures. Sigmoid fitting for Paclitaxel-dose dependent cell viability inhibition suggested an IC50 of 0.21μM for OVCAR8 mono-culture, 1.64μM for HUVECs mono-culture and 2.73μM for the co-culture of HUVECs with OVCAR8, with the co-culture model having the highest IC50 among the three, suggesting a higher drug resistance. Interestingly, as shown in Fig 8(A) for Cisplatin, HUVECs mono-culture did not show an obvious response, such as tubule structure interruption compared with co-culture of HUVECs with OVCAR8, whereas, for Paclitaxel, the HUVECs mono-culture model showed a significant reduction in tubule areas. This lack of effect of Cisplatin on HUVECs cultured alone was also reflected in the tubule areas analysis which is shown in Fig 7(B).

It is noticeable that Cisplatin and Paclitaxel had different dose-dependence patterns on HUVECs cultured in the Matrigel sandwich. Cisplatin showed significant toxicity from concentrations as low as 1nM, while for Paclitaxel this inhibiting effect was lower than Cisplatin at the same concentration. However, with the increase in concentration, the toxicity of Paclitaxel on HUVECs increased at a higher rate than the Cisplatin, until at concentration 1μM the inhibition effect of Paclitaxel started to exceed Cisplatin. Between 1μM and 10μM, the inhibition percentage of Paclitaxel increased from 38.10% to 74.01%, while the inhibition percentage of Cisplatin only increased to 43.76% at 10μM. For OVCAR8 cultured alone in the Matrigel sandwich, the inhibition percentages of Cisplatin and Paclitaxel overlapped from 10nM to 100nM, with the inhibition changes from about 18% at 10nM to 30% at 100nM. However, Paclitaxel showed a significant higher inhibition on OVCAR8 at concentration 1μM, with the percentage as 74.97%, while the inhibition percentage of Cisplatin at the same concentration was only 39.01%. This difference was even bigger at 10μM, with the inhibition percentage of Paclitaxel as high as 88.86% whereas Cisplatin only caused 40.38% viability inhibition of OVCAR8 in the Matrigel sandwich. The effect difference of Cisplatin and Paclitaxel is also reflected in the morphology of HUVECs in the Matrigel sandwich. As shown in Fig 8, there was barely tubule structure change with the increase of the dosage of Cisplatin, while there was dose-dependence tubule structure interruption by Paclitaxel. In the above results, it is noticeable that morphology change can be well consistent with viability inhibition of the drug, such as in terms of HUVECs treated with Paclitaxel, or OVCAR8 treated by Paclitaxel, but this was not always the case. For example, in the HUVECs treated by Cisplatin, though there was a dose-dependence viability inhibition, the tubule structure change was hardly visible even at the highest concentration of 10μM. The response of morphology change could also be more sensitive than viability change, as a significant area decrease was observed OVCAR8 treated by Cisplatin from 100nM to 1μM, which was not reflected well in the viability assay.

Further image analysis with regard to angiogenesis, represented by segmented tubule areas, was carried out to evaluate the responses of the co-culture model to Cisplatin and Paclitaxel, and the comparison between co-culture and endothelial cells cultured alone in sandwich culture is shown in Fig 7(B). For Cisplatin, the DNA interfering drug, the tubule areas shown in Fig 7(B) suggested a dose-dependent manner for the decrease of the tubule areas for the co-culture. For the same concentration of Cisplatin, there was no statistical significance between HUVECs mono-culture and co-culture, though the responses of HUVECs mono-culture were lower than those of co-culture at 1μM. Compared with Cisplatin, the angiogenesis-inhibition effect of Paclitaxel was more significant, as shown in Fig 7(B); 10nM Paclitaxel caused the tubule areas to decrease significantly from 416524 μm^2^ to 296960 μm^2^ (p = 0.028 by Student t-test). In contrast, no statistically significant inhibitory effect was shown in HUVECs mono-culture. It is widely known that Cisplatin is a suitable drug to treat ovarian cancer. The lack of response in the mono-culture HUVECs compared with co-culture shows the limitation of mono-culture and demonstrates that co-culture of HUVECs and cancer cells can truly reveal the anti-angiogenesis potential of a compound in the case of Cisplatin.

## Discussions

Our results suggest the maintaining effect of cancer cells on the tubule structures formed by endothelial cells and our model is more suitable for long-term testing (longer than 72 hours) compared with the traditional angiogenesis assay on the Matrigel. It has been widely accepted that HUVECs are able to form tubule structures on the top of the Matrigel but these tubules only lasted for up to 72 hours. When embedded into the Matrigel, endothelial cells can potentially survive longer but they lose the ability to form tubule-like structures (Ingthorsson, et al. 2010). Our results show that, in 3D sandwich culture, endothelial cells kept their tubule forming ability in a 3D environment (with the whole network thickness around 200μm as shown in Fig 3) and that these tubule networks could be maintained for up to 10 days in co-culture with cancer cells. HUVECs mono-culture in the same sandwich Matrigel structure failed to maintain the tubule structures for more than 48 hours, demonstrating the maintenance effect of cancer cells on those endothelial cell formed tubule structures. This is supported by previous reports that cancer cells have stimulating effects on the proliferation, migration and tubule formation of endothelial cells, by up-regulating growth factors such as VEGF and integrins—the former of which stimulates the growth of endothelial cells and the latter contributes to endothelial cell differentiation in tubule formation [17]. The increase in spheroid area arising from cancer cell proliferation, based on the morphology of the 3D culture, still remained integral until day 10, as spheroids with a significant apoptotic population of cells would have shown a significant morphological change including scattering of cell debris and more irregular spheroid sizes, such as those shown in Fig 8 after treatment with Cisplatin and Paclitaxel. Nevertheless, it is possible that once the spheroids reach a diameter of more than 200μm, due to the limitation of oxygen and nutrient supply, apoptosis could potentially be induced in the central area of the 3D spheroids [18].

The morphology of the co-culture model in this study not only features tubule formation by endothelial cells but also allows the formation of 3D cancer cell spheroids, which is a 3D *in vitro* model widely accepted by other researchers [12]. Many researchers have pointed out the similarities of those cancer cell spheroids to *in vivo* cancerous tissue. These structures were able to be formed due to the 3D structure signals restored by the Matrigel, the laminin-rich extracellular matrix. Signal pathways, like β1-integrin and epidermal growth factor receptor (EGFR), function in a parallel way in traditional 2D culture which requires blocking both pathways at the same time in order to reduce cancer cell growth to below 20% of the control with no down-regulation of expression of either β1-integrin or EGFR, while in 3D they can be integrated in a reciprocal way in which inhibiting either of those two ways is enough to induce growth-arrest and down-regulation of both β1-integrin or EGFR [12, 18]. By applying the contour detected cell segmented image analysis technique, the morphological information of the spheroids, such as spheroid areas, can be separated from the bright field images of the co-culture model to be analysed. This methodology will potentially allow a more efficient analysis of the complex co-culture system without tedious fluorescence labelling which can also cause information loss during long time processing.

Although dose-dependent responses of Cisplatin and Paclitaxel have been studied in 2D models in other researchers’ work, anti-cancer drug testing on 3D cancer models or endothelial cells is still limited. To evaluate angiogenesis regulating effects quantitatively, two measurements were tested in this study—tubule areas and branch points, adapted from angiogenesis parameter profiles built up by previous research [6]. Our results indicate that, compared with Cisplatin, Paclitaxel has more significant inhibitory effects on the angiogenesis abilities of the co-culture model. Supporting evidence for this result is evident in previous research, including the official database of the National Cancer Institute (NCI). Based on the NCI database, Cisplatin and Paclitaxel have similar IC50 on the OVCAR8 cell line (Cisplatin 2.5 μM, Paclitaxel 1.0 μM) [19] but the IC50 difference of these two drugs on HUVECs is about 2500 times: after 24 hours of treatment, the IC50 of Cisplatin on HUVECs is around 50μM [20] while the IC50 of Paclitaxel on HUVECs is 2nM [21]. In other words, responses of HUVECs to those two drugs are very different, though they have similar cytotoxicity on OVCAR8. This demonstrated that compared with the mono-culture model of OVCAR8, co-culture with HUVECs can not only give information on cancer cell response but can also provide extra information on the anti-angiogenesis effects of the tested drugs. One potential explanation for this difference in the anti-angiogenesis effects of Cisplatin and Paclitaxel is their different acting mechanisms. Paclitaxel interferes with the dynamics of microtubules which participate spindle formation in cell mitosis, thus blocking cell proliferation effects [22]. Cisplatin, on the other hand, impairs DNA by interacting with purine bases to promote the formation of DNA crosslinks [23]. Previous studies have pointed out the particular dependence of endothelial cell functions on cytoskeleton dynamics, including proliferation, migration and other steps in the angiogenesis process [21–22] which may explain the stronger inhibition effects of Paclitaxel on HUVECs as shown in Figs 7 and 8. It is noticeable that even HUVECs alone had very low viability following 24 hours in culture. When cultured with OVCAR8 the effect of HUVECs on the response of the co-culture model to the drug tested was still significant. This suggests that the mutually beneficial relationship between endothelial cells and cancer cells that maintains the 3D structure can be potentially enhanced by a 3D culture environment compared with conventional 2D culture. For instance, previous research reported that expression of interleukin 8 (IL-8), an important chemokine for angiogenesis, was increased 17 fold in 3D culture compared with 2D culture [24]. The angiogenesis promoting effect of 3D culture will in return induce the growth and malignancy of cancer cells and thus will increase the drug resistance of cancer cells.

## Conclusions

In summary, the highlights of this study include: 1. Relative long-term co-culture (10 days) was established for the co-culture of HUVECs and cancer cells; 2. An image analysis protocol was developed to provide quantitative information of both 3D tumour growth (spheroid areas) and angiogenesis morphology assays (tubule lengths and areas); 3. In morphology analysis for drug testing, the developed sandwich co-culture model revealed the anti-angiogenesis potential of Cisplatin, while HUVECs mono-culture failed to predict the effect, demonstrating the advantages of co-culture over mono-culture in assays of angiogenesis. However, there are still some limitations of this 3D sandwich co-culture model: 1. The complicated *in vivo* absorption and distribution process of the drugs tested in this study (Cisplatin and Paclitaxel) could not be modelled; 2. The scaffolding material, Matrigel, was anticipated to influence the cell growth and behaviours because it contains many growth factors, such as basic fibroblast growth factor (bFGF) and epidermal growth factor (EGF), so extra caution should be taken when interpreting the data based on a Matrigel system [25]. Based on the above limitations, our future study will focus on: 1. Co-culture will include fibroblasts or macrophages to mimic the real tumour angiogenesis microenvironment to a better level; 2. Well-defined scaffolding materials will be developed, which can provide precisely controlled biological cues for cells.

## Supporting information

S1 Fig A) Morphology of OVCAR8 grown in EBM-2 supplemented with 2% FBS (a) compared with those grown in DMEM supplemented with 10% FBS (b); (B) Viability comparison based on AlamarBlue assay. There is no significant difference between the two groups.
(TIF)
