# Supplementary figures and images for: Morphological analysis of human umbilical vein endothelial cells co-cultured with ovarian cancer cells in 3D: An oncogenic angiogenesis assay
Source: PLoS One. 2017 Jul 3;12(7):e0180296. doi: 10.1371/journal.pone.0180296 (PMC5495474; doi:10.1371/journal.pone.0180296)

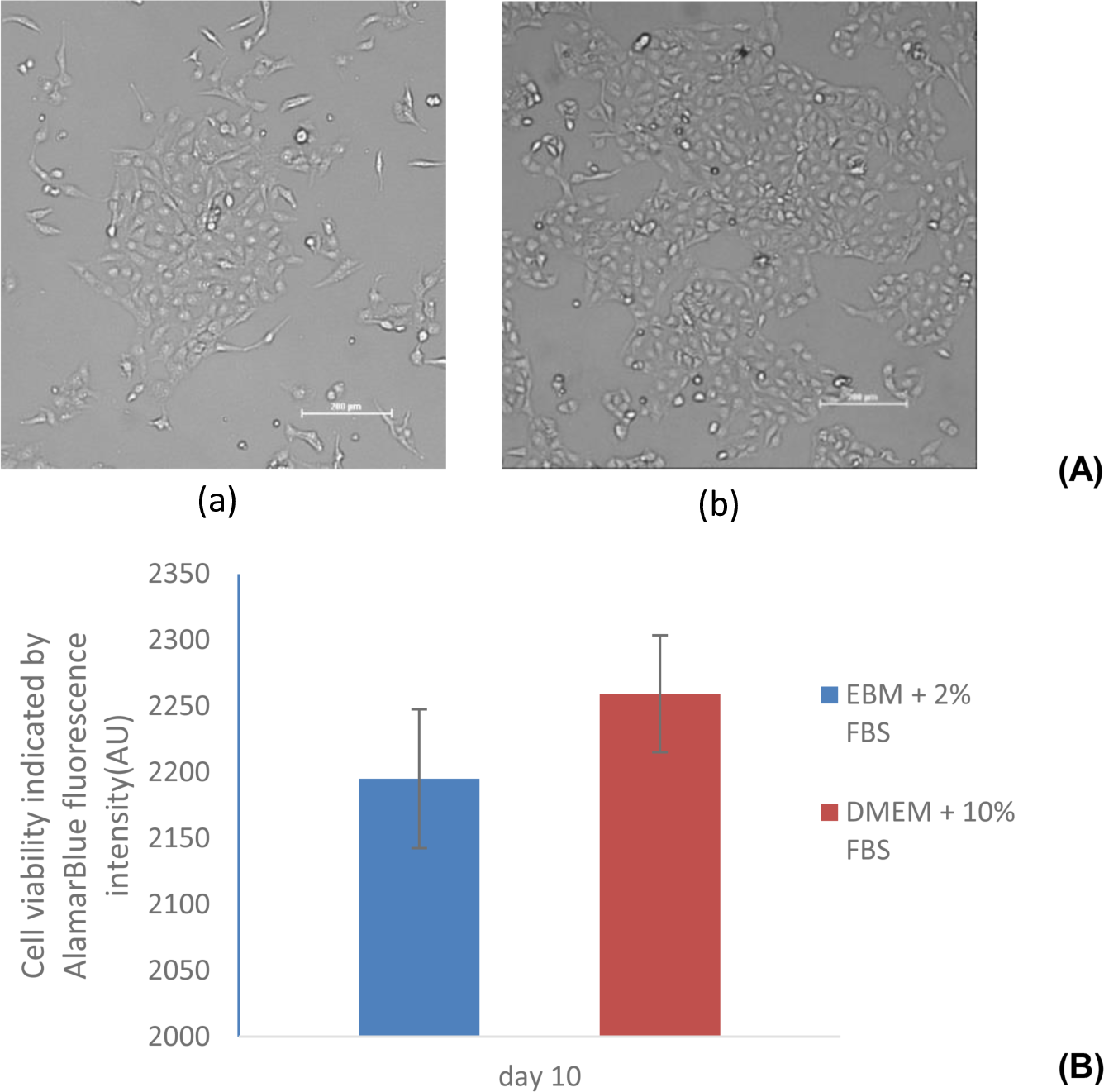

Supplement: S1 Fig — A) Morphology of OVCAR8 grown in EBM-2 supplemented with 2% FBS (a) compared with those grown in DMEM supplemented with 10% FBS (b); (B) Viability comparison based on AlamarBlue assay. There is no significant difference between the two groups. (TIF) [file pone.0180296.s001.tif]
